# Light Chain Multiple Myeloma Presenting As Secondary Cutaneous Amyloidosis: A Case Report on an Uncommon Systemic Manifestation

**DOI:** 10.7759/cureus.48346

**Published:** 2023-11-06

**Authors:** Sérgio P Domingos, Beatriz G Cabrita, Mauro S Siqueira, Patrícia R de Oliveira, Rúben Margaço

**Affiliations:** 1 USF Fonte Luminosa, Administração Regional de Saúde de Lisboa e Vale do Tejo, Lisbon, PRT; 2 USF São João dos Loios, Administração Regional de Saúde de Lisboa e Vale do Tejo, Lisbon, PRT

**Keywords:** multidisciplinary care approach, cutaneous manifestations of multiple myeloma, immunoglobulin light-chain amyloidosis, primary health care, light chain multiple myeloma, extramedullary multiple myeloma

## Abstract

Light chain multiple myeloma presenting as secondary cutaneous amyloidosis is an uncommon systemic manifestation, posing diagnostic challenges. We present a case of an elderly woman with a history of hemorrhoidal disease, who sought medical attention for what she thought was rectal bleeding. Initial examination revealed an ulcerative vulvar lesion. After extensive evaluation by different medical fields, two skin and a bone marrow biopsies, the diagnosis was finally confirmed. This case emphasizes interdisciplinary collaboration, comprehensive evaluation, and awareness of rare multiple myeloma manifestations. It highlights the importance of considering systemic implications even in localized presentations.

## Introduction

Multiple myeloma is a hematological malignancy characterized by the proliferation of plasma cells that accumulate in bone marrow, leading to bone destruction and marrow failure. It accounts for about 1% of all cancer cases and is most frequently diagnosed among people aged 65 to 74 years, with the median age being 70 years. It has an incidence of approximately 4.5 to 6 cases per 100,000 people, per year [1,2]. Multiple myeloma evolves from an asymptomatic, precursor condition, either monoclonal gammopathy of undetermined significance (MGUS) or smoldering multiple myeloma [2,3]. It commonly presents with bone lesions, anemia, hypercalcemia, and renal dysfunction [2-7]. However, it can manifest in various atypical ways, leading to diagnostic challenges [1,5].

Most common causes of vulvar lesions typically diverge from this hematologic disorder, since they often arise from infections, dermatoses, effects of hormonal and systemic disturbances, vulvar intraepithelial neoplasia, and invasive cancer [8]. These causes highlight the diverse array of pathologies that can contribute to vulvar health concerns. Vulvar amyloidosis as a presentation of multiple myeloma is an exceedingly rare phenomenon, with only two documented cases in the existing literature to the best of our knowledge [9,10]. While the rare instances in which myeloma-related vulvar manifestations arise add complexity to the differential diagnosis, they remain outliers in the broader landscape of vulvar pathology [9].

These situations emphasize the pivotal role a family physician assumes when confronted with atypical lesions, regardless of the assumptions patients might have regarding the cause of their condition [3,4,11]. We report a rare case of light chain multiple myeloma presenting as secondary cutaneous amyloidosis in an elderly woman with a history of hemorrhoidal disease.

## Case presentation

A 78-year-old woman with a medical history of hypertension, dyslipidemia, ischemic heart disease, and hemorrhoidal disease, schedules an urgent appointment with her general practitioner for recurring rectal bleeding. Approximately three months prior, the patient had attended a medical appointment where she reported experiencing rectal prolapse and rectal bleeding starting a month earlier. She had declined a physical examination due to the spontaneous resolution of her symptoms and had also refused to undergo the recommended complementary exam - a colonoscopy. Additionally, routine analyses were ordered, which later revealed a mild anemia with hemoglobin levels of 10.2 g/dL.

**Figure 1:**
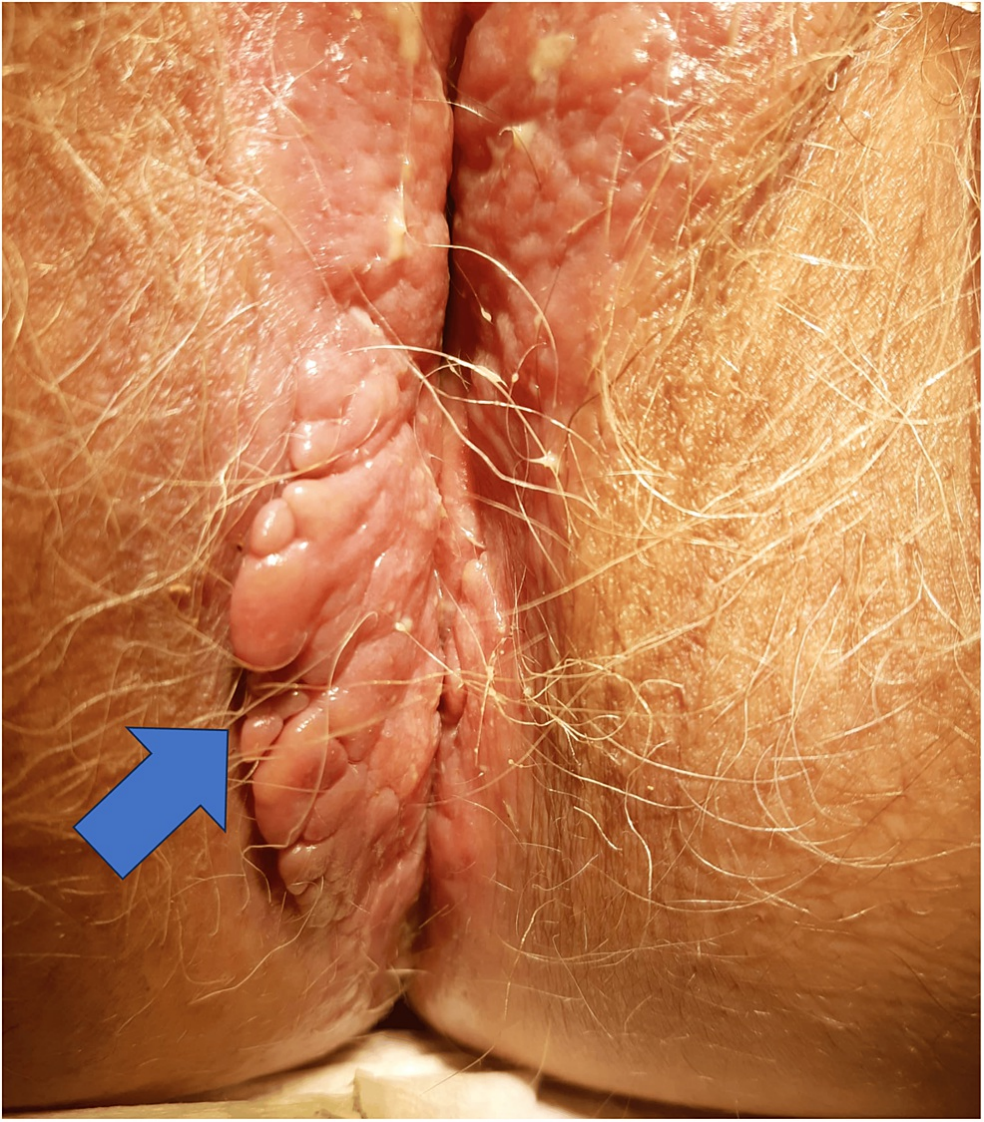
Irregular exophytic vaginal lesion

Given her personal history of hemorrhoidal disease, the patient had attributed her previous and current symptoms - namely, mild fatigue in addition to the aforementioned rectal bleeding - and analytical changes to this condition. However, on the present appointment, physical examination revealed an irregular exophytic vaginal lesion extending to the perianal region (Figure 1).

Due to the extensive nature and atypical appearance of the lesion, the patient was urgently referred to the gynecology department, where a vulvoscopy was performed, along with a biopsy of the lesion and pelvic MRI. This revealed an irregular exophytic lesion that occupied the entire posterior half of the right labia majora, extending across the perineal area to the perianal region, involving a portion of the left labia majora, with areas of erosion.

The histological report described skin showing signs of hyperkeratosis, slight epidermal atrophy, and significant dermal hyalinization, without inflammatory infiltrate, dysplasia, or invasive neoplastic tissue. Additionally, the MRI showed no signs of involvement of adjacent structures or systemic disease. These features were consistent with atrophic lichen sclerosus and, as such, the patient was referred to a dermatologist for further specialized care.

During this consultation, considering the physical examination and complementary tests, it was concluded that, while suggestive, the final diagnosis might not be as straightforward and that other possibilities, namely, the presence of hematological or autoimmune disease, should first be excluded. Consequently, further exams were conducted, including protein electrophoresis and antibody serum levels, as well as a new skin biopsy for staining and immunohistochemical analysis.

This biopsy revealed an amorphous eosinophilic material deposit throughout the thickness of the dermis, predominantly in a perivascular location. This was consistent with amyloid substance (positive Congo Red staining) and compatible with an amyloid plaque. Immunohistochemical analysis of the skin slide confirmed the presence of light-chain amyloidosis. This, in conjunction with the protein electrophoresis revealing an increase in lambda chains, resulted in a final diagnosis of light chain multiple myeloma with an atypical gynecological presentation. Subsequently, the diagnosis was further validated through a bone marrow biopsy.

## Discussion

The presented case highlights several crucial aspects of clinical practice, shedding light on the complexities and challenges that can emerge when dealing with atypical clinical presentations [4]. Firstly, the importance of conducting a thorough physical examination as part of a full evaluation cannot be overemphasized [3,4]. In this case, the patient’s medical history, marked by hemorrhoidal disease, led her to attribute her symptoms solely to that condition, downplaying the distinctive appearance of the lesions. This is a stark reminder that subjective complaints should always be considered alongside objective findings and not as an isolated finding, in order to arrive at an accurate diagnosis. 

Moreover, the fact that the lesions emerged within the gynecological system should not limit the perspective to assuming a purely localized issue. Medical professionals must remain alert to the possibility of systemic conditions manifesting in unexpected ways. This case serves as a reminder that seemingly isolated gynecological symptoms can be fragments of a larger systemic puzzle, necessitating a comprehensive approach to diagnosis and treatment. The case also emphasizes the value of a multidisciplinary approach [5]. The importance of collaboration between specialties can be abundantly found in the literature [6,12,13]. In this case, each field provided a unique insight contributing to a more accurate diagnosis and personalized treatment plan.

We would also like to highlight how, even if a test result does not instantly affirm the suspected diagnosis, it should not be immediately discarded. Instead, it should be integrated into the broader clinical picture to steer further investigations and decisions.

Lastly, the case serves as a poignant reminder that considering uncommon presentations is vital when more common causes have been ruled out [4]. Clinicians should retain an open-minded stance and be ready to explore less frequently encountered possibilities. As illustrated by this case, seemingly disparate symptoms and clinical presentations can at times converge into a rare but consequential diagnosis that might otherwise go unnoticed.

## Conclusions

This case sheds light on the complex landscape of atypical myeloma-related vulvar manifestations within the broader context of gynecological pathology. While multiple myeloma is often characterized by more conventional clinical features, this case underscores the diagnostic challenges posed by its ability to manifest atypically. While the unique presentation, in this case, adds complexity to the diagnostic journey, it serves as a reminder that even in the face of atypical manifestations, clinicians must remain vigilant and comprehensive in their approach, integrating interdisciplinary collaboration, objective signs, and a holistic patient-centered perspective to accurately identify and address such rare occurrences. In summary, this case calls attention to the importance of documenting atypical presentations and to the dynamic nature of medical practice, urging healthcare professionals to exercise clinical acumen, embrace multidisciplinary collaboration, and remain vigilant for atypical presentations, all while ensuring thorough and patient-centered care.
